# Initial Genomics of the Human Nucleolus

**DOI:** 10.1371/journal.pgen.1000889

**Published:** 2010-03-26

**Authors:** Attila Németh, Ana Conesa, Javier Santoyo-Lopez, Ignacio Medina, David Montaner, Bálint Péterfia, Irina Solovei, Thomas Cremer, Joaquin Dopazo, Gernot Längst

**Affiliations:** 1 Department of Biochemistry III, University of Regensburg, Regensburg, Germany; 2 Department of Bioinformatics and Genomics, Centro de Investigación Príncipe Felipe, Valencia, Spain; 3 Department of Biology II, Ludwig-Maximilians University of Munich, Planegg-Martinsried, Germany; Max-Planck-Institute of Immunobiology, Germany

## Abstract

We report for the first time the genomics of a nuclear compartment of the eukaryotic cell. 454 sequencing and microarray analysis revealed the pattern of nucleolus-associated chromatin domains (NADs) in the linear human genome and identified different gene families and certain satellite repeats as the major building blocks of NADs, which constitute about 4% of the genome. Bioinformatic evaluation showed that NAD–localized genes take part in specific biological processes, like the response to other organisms, odor perception, and tissue development. 3D FISH and immunofluorescence experiments illustrated the spatial distribution of NAD–specific chromatin within interphase nuclei and its alteration upon transcriptional changes. Altogether, our findings describe the nature of DNA sequences associated with the human nucleolus and provide insights into the function of the nucleolus in genome organization and establishment of nuclear architecture.

## Author Summary

It is becoming increasingly clear that the nuclear organization and location of genes in metazoan organisms is not random. Functionally related genes are often found next to each other in the linear genome, and distant DNA elements or DNA regions residing on different chromosomes may reside in specific nuclear compartments. The largest nuclear compartment is the nucleolus with its shell of perinucleolar DNA. The nature of the nucleolus-associated DNA, the targeting mechanism, and the cellular function of this subset of genomic DNA are not known. In the present study we report for the first time the high-resolution analysis of a nuclear compartment by sequencing, microarray analysis, and single-cell analysis. We have characterized the nucleolus-associated DNA on sequence level and by 3D microscopy and have determined common elements and the molecular function of this compartment.

## Introduction

The largest and densest nuclear compartment is the nucleolus with its shell of perinucleolar DNA. The nucleolus is a unique object to study genome activity, since all three RNA polymerases are involved in the highly dynamic and tightly regulated ribosome biogenesis process, which is its main function. High proliferation activity of tumour cells coincides with high ribosome biogenesis activity thus exposing the nucleolus as a promising target in cancer therapy [1]. In addition, cell-type and function-dependent nucleolar localisation of tumour suppressor proteins, such as p53, MDM2 or p14ARF indicates the role of the nucleolus in carcinogenesis [2]–[5]. A number of other biological processes (e.g. senescence, RNA modification, cell-cycle control and stress sensing) are also regulated in the nucleolus and connect it to several functional networks of the cell [2]–[7]. Furthermore, chromatin motion is constrained at nucleoli or nuclear periphery, and disruption of nucleoli increases motility of chromatin domains, indicating the role of the nucleolus in higher-order chromatin arrangement [8]. The nucleolus can therefore be considered as a well-suited model system to investigate functional consequences of genome organisation. It is less well known, however, that alteration in the nucleolus might be linked to multiple forms of human disease, including viral infections. The interaction between viruses and the nucleolus is a pan-virus phenomenon, which is exhibited by DNA viruses, retroviruses and RNA viruses [9],[10]. Moreover, multiple genetic disorders have been mapped to genes that encode proteins located in nucleoli under specific conditions. These include Werner [11], fragile X [12],[13], Treacher Collins [14], Bloom [15], Rothmund–Thomson [16] and dyskeratosis congenita syndromes [17] and Diamond-Blackfan anemia [18].

Nucleoli are easily detectable under the microscope, however, despite the simple methods of nucleolus isolation, their molecular structure is largely unknown. The nucleolar proteome has been recently analysed by high-throughput mass-spectrometry [19], but the nucleic acid composition of nucleoli had not yet been determined. Therefore the aim of our investigations was to construct and characterize the first high-resolution, genome-wide map of NADs. Recent advances in sequencing and microarray technologies provided excellent platforms to subject nucleolus-associated DNA (naDNA) to critical scrutiny. The results presented here help to understand the mechanisms of nuclear information packaging by macromolecular assemblies and the functional compartmentalisation of the nucleus.

## Results/Discussion

**Figure 1:**
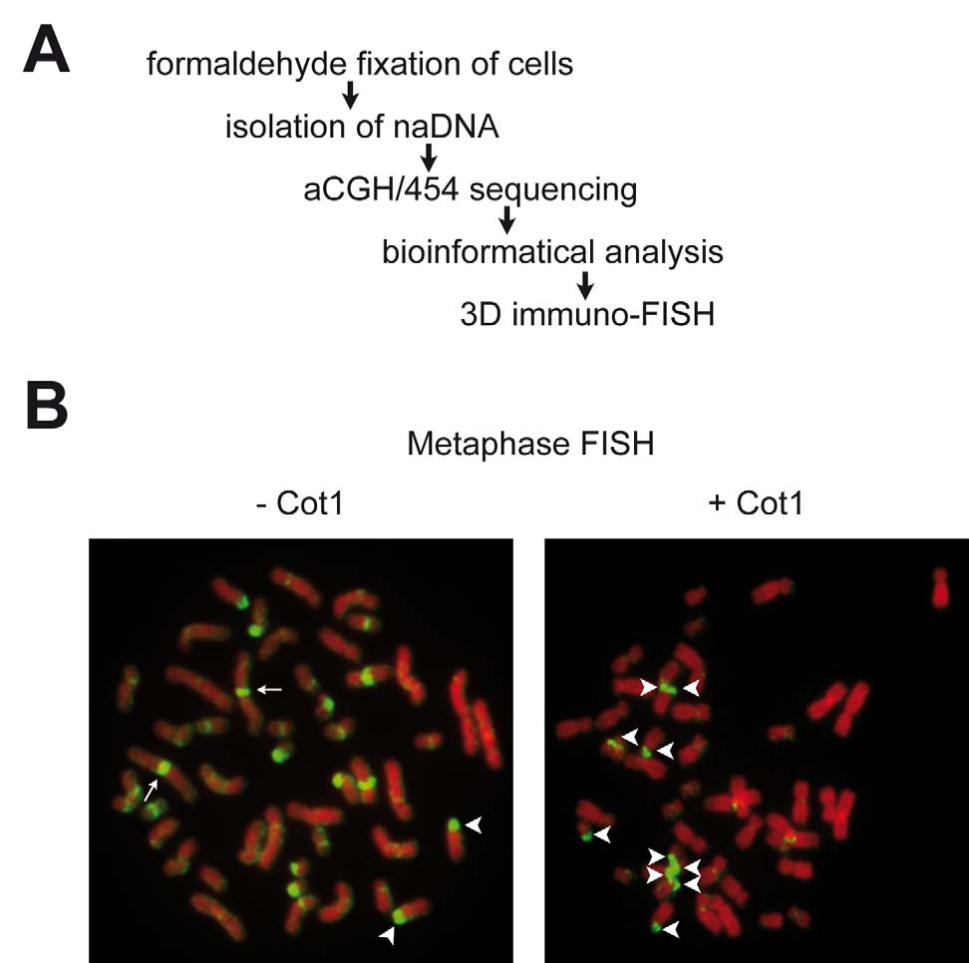
Genome-wide analyis of nucleolus-associated DNA. (A) Experimental strategy. (B) 2D FISH analysis of nucleolus-associated DNA on human female lymphocyte metaphase spreads in the absence (-Cot1) or presence (+Cot1) of Cot1 competitor DNA. Arrows indicate chromosome 1 centromeres, arrowheads indicate p-arms of acrocentric chromosomes.

**Figure 2:**
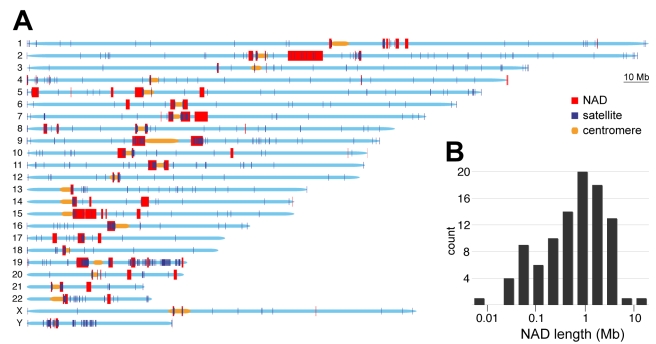
Genomic and size distribution of NADs. (A) Distribution of NADs together with satellite repeats along human chromosomes. Note that the p-arms of the five acrocentric chromosomes (13, 14, 15, 21 and 22) were not analysed because they are not assembled in the hg18 genome build. NADs are labeled with red, satellite repeats with deep blue, centromeres with yellow and chromosomes with light blue (B) Histogram of NAD sizes; median  = 749 kb; a total of 97 NADs were identified.

**Figure 3:**
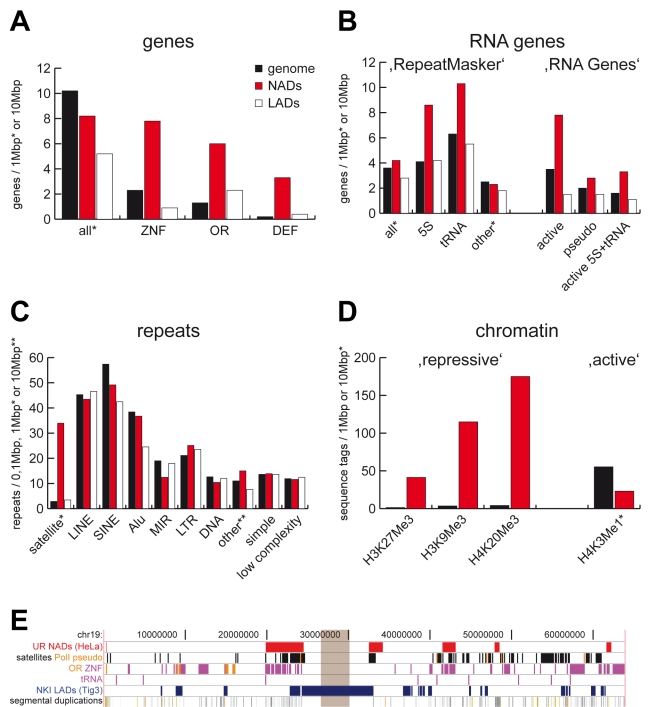
Sequence and chromatin features of NADs. (A) RefSeq gene (B) RNA gene and (C) repeat statistics of NADs, genome and LADs. ZNF, OR and DEF indicate zinc finger, olfactory receptor and defensin gene families, respectively. RNA gene analysis of the ‘RepeatMasker’ and ‘RNA Genes’ tracks of the UCSC Genome Browser are shown on the left and right, respectively. (D) Chromatin features of NADs. Enrichment of functionally characterised repressive histone marks H3K27Me3, H3K9Me3 and H4K20Me3 in NADs are shown on the left, whereas depletion of the active histone mark H3K4Me1 is shown on the right. Genome, NADs and LADs values are labeled uniformly in (A–D) with black, red and white, respectively. The complete analysis is summarised in Table S5 and S6. (E) NADs and their typical genomic features on chromosome 19. Brown rectangle indicates the centromere. Abbreviations: UR (Universität Regensburg) NADs – nucleolus-associated chromatin domains identified in this study, PolI pseudo –pseudogenes of RNA polymerase I transcribed rRNA genes, OR – olfactory receptor genes, ZNF – zinc finger genes, tRNA – transfer RNA genes (and pseudogenes) transcribed by RNA polymerase III, NKI (Nederlands Kanker Instituut) LADs–lamin-associated chromosome domains identified in the Tig3 cell line [21].

Because the nucleolar proteome was analysed in HeLa cells [19], our study started with the purification of nucleoli from this widely used model system (Figure 1A). Enrichment of the nucleolar transcription factor UBF and depletion of nuclear lamina proteins laminA/C from the nucleolar fraction was monitored by Western blot. Nucleolus-associated DNA was then isolated, and ribosomal DNA (rDNA) enrichment was measured by quantitative PCR (Figure S1). To analyse the genomic localisation of purified naDNA at low resolution, we performed 2D FISH experiments. Hybridisation of naDNA on human lymphocyte metaphase spreads shows that it appears predominantly on p-arms of acrocentric chromosomes, the location of the repetitive rDNA, and on centromeres of several chromosomes. The addition of the repetitive Cot1 competitor DNA suppresses binding of the naDNA probe to various chromosomal regions, but not to rDNA-containing nucleolar organiser regions (NORs). The result clearly demonstrates that rDNA, moreover pericentomeric and centromeric repetitive sequences are overrepresented in naDNA compared to other chromosomal regions (Figure 1B). Next, naDNA was analysed using Nimblegen whole genome microarrays at 6,270-bp median probe spacing resolution and compared to genomic DNA by performing two-colour hybridisation (aCGH). The aCGH data reinforced the results of the 2D FISH experiments: p-arm-adjacent regions of the acrocentric chromosomes and pericentromeric regions are enriched in naDNA. More interestingly, many other chromosomal regions are also present in the naDNA fraction (Figure 2A, Figure S2 and S3). For example, a large part of chromosome 19 associates with the nucleolus (Figure 3E). This finding explains the presence of chromosome 19 in central regions of the interphase nucleus [20], being close to the nucleoli. To elucidate NAD-specific sequence signatures in more detail, 454 sequencing was performed. In total 47,378,399 bases were sequenced in 218,030 reads with an average length of 217 bases/read. We used the complementary set of microarray and sequencing data to visualise the genome-wide localisation of NADs. Genome-wide studies are performed almost exclusively using one high-throughput strategy, which limits the quality of the detection. The combination of techniques compensates the inherent mistakes of the different methods. Our results clearly show that certain NADs are detectable only with one of these approaches (Figure S2 and Table S1). It is important to mention that the p-arms of the five acrocentric chromosomes, which contain rDNA and satellite repeats, are not represented in the hg18 genome build and, therefore, were not included in our analysis. In addition to the previously described pericentromeric locations, a significant number of the NADs (nine) localised in sub-telomeric regions. Altogether, 97 chromosomal regions that are associated with nucleoli were identified, encompassing about 4% (126,217,765 bp) of the genome. Our study detected the most frequent nucleolus-associated chromosome domains using stringent cut-off parameters for domain definition (Figure 2A, Figure S2 and S3, Table S1, and Materials and Methods).

After genome-wide NAD identification, sequence and chromatin features were compared to the whole genome and lamina-associated domains (LADs). LADs were recently determined by high-resolution mapping using DamID technology [21]. The size distribution (0.1–10 Mb) and median sequence length (749 kb) of NADs (Figure 2B) were similar to LADs (0.1–10 Mb, 553 kb) suggesting that the architectural units of chromosome organisation within the mammalian interphase nucleus are about 0.5–1 Mb in length.

One thousand thirty-seven genes have been identified within NAD sequences according to the RefSeq gene database, 729 of which were non-redundant (Table S2). Surprisingly, certain gene families were frequently associated with the nucleoli, even though the overall gene density in NADs is about 20% lower than in the whole genome. We observed a 4-fold enrichment of zinc-finger (ZNF) genes in NADs compared to the genome. Olfactory receptor (OR) and defensin genes were enriched in both NADs and LADs, but the enrichment was far greater in NADs (Figure 3A). Moreover, two of the six large clusters of immunoglobulin and T-cell receptor genes [22] overlap with NADs, and one other is juxtaposed to a NAD (Figure S3). The gene families mentioned above have two common features: their members are in large gene clusters, and they are expressed in a tissue-specific manner. This phenomenon suggests that these large chromosomal regions may change their sub-nuclear position with regard to their transcriptional activity. In addition, both immunoglobulin and OR genes exhibit monoallelic expression [23],[24]; therefore, nucleoli may be involved in this type of gene regulation. Though, this has to be tested for each individual gene in specific model systems. Besides the response to other organisms and odour perception, additional biological processes and molecular functions are specifically associated with genes localised in the vicinity of the nucleolus, including tissue development and embryo implantation. (Figure S4 and S5 and Table S3). Carcino-embryonic antigen cell adhesion molecule (CEACAM) genes and pregnancy-specific glycoprotein (PSG) gene clusters, whose protein products regulate implantation, were also found next to and within NADs, respectively. Additionally, a large number (119) of small nucleolar RNA (snoRNA) genes were identified within one NAD on chromosome 15. However, this association may be explained by the close proximity of this cluster to the rDNA repeats (distance of 5 Mb).

RNA genes located within NADs were characterized using the datasets of the ‘RepeatMasker’ and ‘RNA Genes’ databases of the Genome Browser. Both analyses show that 5S and tRNA genes, both of which are transcribed by RNA polymerase III, are specifically enriched in NADs but not in LADs. In contrast, other RNA genes are distributed with a similar frequency in NADs and the rest of the genome (Figure 3B). This finding proofs that RNA polymerase III-transcribed genes co-localise with nucleoli [25]–[27], which is the site of RNA polymerase I transcription. These observations suggest that spatial regulation may play a role in coordinated, well-tuned transcription of the RNA components of the protein translation machinery.

Analysis of the repetitive elements showed a more than 10-fold enrichment of satellite repeats in NADs and depletion of SINE - especially MIR–repeats (Figure 3C). We next performed a detailed quantitative analysis of all major satellite repeat subclasses located within NADs. (Figure S6). Our results demonstrate that the major building blocks of NADs are the alpha-, beta- and (GAATG)_n_/(CATTC)_n_-satellite repeats, whereas other types of satellite repeats (e.g. MSR1, D20S16, SATR2) were depleted. These data confirm and extend previous studies [28],[29] that describe nucleolar association of satellite repeats, but do not analyse them in detail. Taken together with the fact that D4Z4 macrosatellite repeats are located on the short arms of acrocentric chromosomes [30] and that ‘RepeatMasker’ does not contain information about low copy number repeats (e.g., segmental duplications or macrosatellites), we extended our investigations to such repetitive elements and showed that these genomic features are enriched in NADs (Figure S3 and Table S4). The presence of low-copy number repeats in NADs underlie the difficulties of alignment-based localisation of naDNA sequences within the genome: segmental duplications and major satellites will be mapped to more than one region [31],[32], thus the nucleolar association of chromosome regions containing such sequences has to be confirmed by neighbouring sequences or in 3D FISH experiments. Enrichment of satellites and segmental duplications in NADs may also explain the assignment of several domains to chromosome Y even though HeLa cells are derived from a female. The Y chromosome has been shown to co-localise with nucleoli in the interphase nucleus [29],[33], indicating that such low-copy number repeats are maybe involved in nucleolar targeting. The detailed map of nucleolus-associated chromosomal regions and genomic features enriched in NADs is shown in Figure 3E for chromosome 19. The complete set of data is shown in Figure S3 and Table S5.

In order to reveal specific chromatin patterns enriched within the nucleolus-associated chromatin domains, we used the genome-wide maps of histone modifications [34]–[36]. Multiple repressive histone marks were specifically enriched, whereas the active histone mark H3K4Me1 was significantly depleted in NADs. As mirrored by the enrichment of repressive histone marks, we observed the reduced global gene expression in NADs (Figure 3D and Table S6). These findings imply that NADs tend to form large inactive chromatin domains in the interphase nucleus. However, nucleolus-associated inactive chromatin differs markedly from lamina-associated inactive chromatin in the kind of repetitive elements and the gene-associated biological processes, suggesting that multiple domains of functionally distinct inactive chromatin exist within the nucleus. Furthermore, the presence of the highly expressed classes of 5S RNA and tRNA genes in nucleolus-associated chromatin indicates that the perinucleolar region is not exclusively transcriptionally silent.

**Figure 4:**
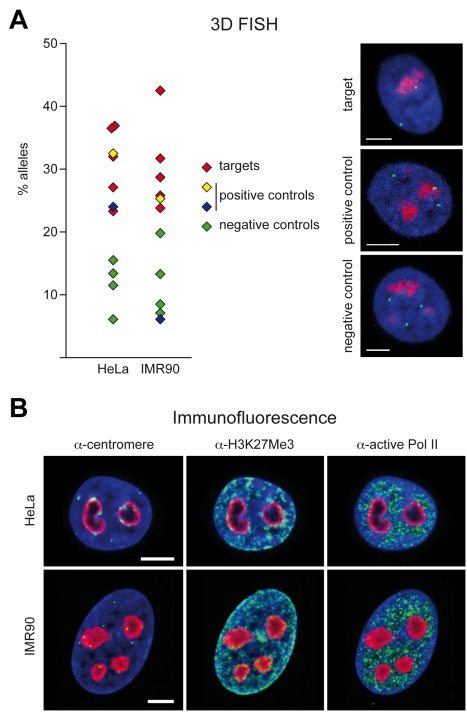
3D immuno–FISH analysis of nucleolus-associated chromatin domains. (A) Histograms show the frequency of the nucleolar localisation of NADs and control chromosomal regions detected by 3D FISH in HeLa cervix carcinoma and IMR90 diploid fibroblast cells. Percentage of nucleolus-associated alleles is shown on the left. Red diamond indicates target, green ones negative controls, whereas yellow diamond indicates the chromosome X pericentromeric and blue diamond the 5S cluster positive controls, respectively (see Table S7 for further BAC details). Single light optical sections of HeLa nuclei are shown on the right. BAC hybridization signals of RP11-90G23 target, RP5-915N17 positive control and RP11-81M8 negative control BACs are shown in green, nucleolar staining in red and DAPI counterstain in blue (scale bars: 5 µm). (B) α-H3K27Me3, α-centromere, α-active Pol II and α-B23/nucleophosmin immunostaining of HeLa and IMR90 cells. α-H3K27Me3, α-centromere and α-active Pol II signals are shown in green, nucleolar staining in red and DAPI counterstain in blue (scale bars: 5 µm).

We used 3D immuno-FISH to confirm whether NADs revealed by the high-throughput methods co-localise with nucleoli. Nucleo li were stained with an α-B23/nucleophosmin antibody, and we have chosen 11 genomic loci that were analysed by appropriate BAC clones. Target, negative and positive control regions were selected from different chromosomes (Table S7, Figure S7, and Materials and Methods). The pericentromeric Xq11.1 region and the 5S rDNA cluster at 1q42.13 served as positive controls [26],[37]. The combination of microarray and high-throughput sequencing analysis revealed a high-fidelity list of nucleolus-associated DNA as all of our selected NADs were more frequently associated with nucleoli of HeLa cells than the negative controls. To prove whether the nucleolar association of these chromosomal regions is a cell type specific feature or it is a general property in human cells, IMR90 embryonic lung fibroblasts were analysed. In contrast to HeLa, IMR90 cells possess diploid karyotype and they are not immortal. Except the 5S rDNA cluster on chromosome 1, all selected regions showed similar levels of nucleolar association in IMR90 and HeLa cells (Figure 4A and Figure S8), suggesting that the nucleolar targeting of certain chromosomal regions is a common feature in human cells.

We next addressed the function of transcription in DNA targeting to the nucleolus by monitoring nucleolus association of selected chromosomal domains upon transcriptional inhibition. We used α-amanitin to block transcription by RNA polymerases II and III, whereas the synthesis of the 47S rRNA precursor was repressed by the addition of actinomycin D. We found that the specific inhibition of any of the RNA polymerases results in spatial reorganization of the nucleolus-associated domains (Figure S9 and Table S7), which indicates that the nucleolus forms a functional unit together with the associated perinucleolar chromatin. However, the concomitant partial disruption of nucleolar structures [38] makes the interpretation of such experiments difficult.

In addition to localisation studies of single chromosomal regions, three typical features of the perinucleolar chromatin were visualised. To this end, five-colour immunofluorescence experiments were performed, which allowed direct comparison of the signal distributions of centromere, H3K27Me3 and active RNA polymerase II localisations in the same cell. RNA polymerase II transcription was depleted around nucleoli, furthermore the frequent association of H3K27Me3 and centromere signals with nucleoli reinforced the results of the bioinformatic analysis of NADs. Both HeLa and IMR90 cells showed similar localisation of these nuclear marks and the observed punctuated patterns suggest that functionally distinct chromatin domains co-exist around nucleoli (Figure 4B and 4C and Figure S10).

We report here the mapping and characterization of nucleolus-associated chromatin domains in the human genome. Bioinformatics and statistical analyses reveal that the main building blocks of NADs are certain types of satellite repeats, tRNA and 5S RNA genes and members of the ZNF, OR, defensin and immunoglobulin gene families. Thus, our data suggest that certain type of satellite repeat sequences play an important role in establishing of NADs. Indeed, the internal scaffold of the nucleolus, the rDNA repeats were analysed only by qPCR (Figure S1), but not in our high-throughput studies for several reasons: i) they are not represented in the hg18 genome build, ii) repetitive sequences are not printed on microarrays, iii) the number of 454 sequencing reads depends on the GC content, which is very variable throughout the rDNA repeat (Figure S11).

The findings of a recent publication indicate that centromeric nucleoprotein complexes may be targeted to the nucleolus via an alpha-satellite RNA-mediated mechanism [39], and address the importance of transcription in this process. These data suggest that transcription has a general regulatory role in maintaining the nuclear architecture around the nucleolus. The transcribed RNA may be bound by nucleolar RNA-binding proteins, which sequester NADs to the nucleolar periphery. On the other hand, our results imply that there is not a unique predictor sequence – in addition to certain satellite repeats, other elements e.g. tRNA genes, 5S RNA genes may be sufficient for the nucleolar targeting of individual chromatin domains. The aforementioned DNA elements, together with specific RNA molecules and scaffold proteins like UBF, may coordinate the (at least partial) self-assembly of the nucleolus with its shell. The principles of the assembly might be similar to the ones that were demonstrated recently for the pseudo-NORs [40],[41] and for the Cajal-body [42], where single DNA, protein or RNA scaffolds were able to nucleate the formation of nuclear compartments. Further experiments are required to uncover the molecular steps of transcription-dependent nucleolar targeting of different groups of NADs and to identify the players in this process. The dynamics of nucleolus association during cell cycle and cell differentiation will be addressed in future studies. The functional organisation of the nuclear architecture is studied intensively [43]–[46] and the identification of NADs in the present work provides a basis for the better understanding of the role of nucleoli in the spatial organisation of the human genome.

## Materials and Methods

### Population average–based analyses

HeLa cervix carcinoma cells were cross-linked with 1% formaldehyde and nucleoli were isolated as described [47]. rDNA content of equal amounts of naDNA and genomic DNA was quantified in real-time PCR reactions.

Oligonucleotide sequences:

Hr132F: 5′CCTGCTGTTCTCTCGCGC,

Hr155P: 5′FAM-AGCGTCCCGACTCCCGGTGC-TAMRA,

Hr198R: 5′GGTCAGAGACCCGGACCC;

Hr9776F: 5′GCCACTTTTGGTAAGCAGAACTG,

Hr9802P: 5′FAM-CTGCGGGATGAACCGAACGCC-TAMRA,

Hr9840R: 5′CATCGGGCGCCTTAACC.

Numbers indicate rDNA (GenBank Acc. No U13369) position relative to the transcriptional start site. Two rDNA regions were measured in technical triplicates from two biological replicate experiments. UBF and laminA/C protein levels were monitored with the sc-9131 and sc-20681 antibodies (Santa Cruz Biotechnology), respectively.

naDNA was isolated and subjected to 454 sequencing (MWG-Biotech) and microarray analysis on HG18 CGH 385K WG Tiling v1.0 platform (Nimblegen). Genomic features of NADs were analysed using the UCSC Table Browser (http://genome.ucsc.edu/cgi-bin/hgTables) and chromatin features using the Ensembl Database (http://www.ensembl.org) and the GSE12889 NCBI GEO dataset. Genomic features were visualised using Galaxy (http://galaxy.psu.edu/) and the UCSC Genome Browser (http://genome.ucsc.edu/). All analyses were performed on the hg18 genome build. Biological processes and molecular functions associated with NAD-located genes were analysed by using FatiGO [48].

Array CGH, 454 sequencing and subsequent data analysis were performed as follows: naDNA samples from two biological replicate experiments were subjected to microarray analysis on HG18 CGH 385K WG Tiling v1.0 platform. Hybridisation and pre-processing of hybridisation signals were performed at Nimblegen. For each of the samples, regions of increased intensity measurements were considered to be relevant if their mean value was greater than the 85 percentile of the sample distribution at 0.1 Mb running window size. Only the intersection of relevant regions across the microarray replicas was considered as a NAD.

High-throughput sequencing was performed using the Roche GS FLX system. One of the aCGH analysed naDNA samples was taken as template for sequencing. 454 sequence reads were quality filtered and automatically assembled into contigs with the Newbler Assembler software at MWG-Biotech. Contigs were matched against the human genome using BLAT. Repeat masked sequences were used both for 454 data and genome data. For matching a 95% of sequence identity and coverage was requested and a maximum gap size of 3 was permitted. Of the mapped reads, 88% had unique hits. 454 data was widely spread on the genome. Only a few regions had higher intensity, mainly around centromeres. For domain detection, 454 data was first transformed into a binary (1/0) signal indicating presence/absence of mapped reads at chromosome positions defined by 100 nts length segments situated at a 1000 nts inter-spacing. A running mean algorithm was run on these data with a window size of 100 (which implies an actual chromosome window size of 0.1 Mb), to identify chromosomal regions with higher abundance of 454 sequencing hits (red bars in plots). 454 ‘Chip-Seq’ domains were selected as those areas with a running mean value above the 98% of the chromosome percentile. This arbitrary threshold fits well visual evaluation of 454 data as well as aCGH data. Finally, 454 regions were edited and border positions were curated manually. The significance of the 454-based NAD determinations was assessed empirically by comparing the number of reads in each of the detected NADs against the distribution of number of reads in 1000 randomly selected same-chromosome regions of the same size. The significance is then obtained as the quartile position of the NAD reads number in the random distribution.

454 and aCGH domains were merged in one single list of NADs. For merging, overlapping regions from both technologies were fused in one domain. Domain borders were defined following aCGH data unless the absence of array probes at merged borders suggested to use the 454 limits. Furthermore, adjacent regions separated by less than 0.1 Mb were joined to single domains.

### Data deposition

Microarray data have been submitted to the ArrayExpress Database (http://www.ebi.ac.uk/microarray-as/ae/) under accession number E-MEXP-2403. 454 sequencing data have been submitted to the Sequence Read Archive (http://www.ncbi.nlm.nih.gov/Traces/sra/) under accession number SRA009887.3.

### Single-cell experiments

2D FISH experiments were performed on HeLa and human female lymphocyte metaphase spreads according to standard protocols. naDNA was labelled without amplification. NAD target and control BACs were selected as follows: RP11-434B14 (Xq11.1; ‘X cen’) and RP5-915N17 (1q42.13; ‘5S’) were used as positive controls. Perinucleolar localisations of the X chromosome and the large 5S rDNA cluster on chromosome 1 were reported previously [26],[37]. RP11-90G23 (8q21.2; ‘REXO1’) and RP11-173M10 (13q21.1; ‘7SK’, encompassing a 7SK RNA gene) were selected based on 454 sequencing data. We tested in the latter case if smaller 454 signals, which have not identified NADs could also be associated with nucleoli. RP11-44B13 (19q13.12; ‘27ZNF’) –selected based on our microarray data - marks a chromosomal fragment in FISH experiments where 27 KRAB-ZNF genes are located. The KRAB-ZNF gene cluster at 19q13.12 represents a SUV39H1 and CBX1 binding region. Our 3D FISH results reveal spatial features of this locus, which was formerly characterized at the level of chromatin domain organisation [49]. RP11-89H10 (3p12.3; ‘FRG2C’) and RP11-413F20 (10q26.3; ‘FRG2B’) were selected from combined aCGH/454 and aCGH results respectively. Both chromosomal regions contain D4Z4 major satellite repeats which may have nucleolar targeting potential. RP11-89O2 (3p14.1; ‘FRG2C ctrl’) and RP11-123G19 (10q24.1; ‘FRG2B ctrl’) served as negative controls for the latter two targets. RP11-81M8 (19p13.3; ‘REXO1’) covers a large 2 Mb chromosome fragment. This region contains the REXO1 gene thus having similarity at the primary sequence level to the REXO1L target and serves as its negative control. The negative control of the ZNF gene cluster (RP11-1137G4; 19p13.3-19p13.2; ‘ZNF557’) contains a single ZNF gene.

3D immuno-FISH experiments were performed as described [50]. In localisation experiments α-B23/nucleophosmin (Sigma, B0556), α-H3K27Me3 (Upstate, 07-449), α-active Pol II (Covance, MMS-129R), α-centromere (Antibodies Inc., 15–134) and different fluorescence dye-conjugated secondary antibodies, furthermore BAC clones RP11-90G23, RP11-173M10, RP11-44B13, RP11-89H10, RP11-413F20, RP11-81M8, RP5-915N17, RP11-1137G4, RP11-89O2, RP11-123G19 and RP11-434B14 were used on HeLa cervix carcinoma cells and IMR90 lung embryonic fibroblasts. HeLa cells were treated with 75 µg/ml or 300 µg/ml α-amanitin for 5 hours in order to inhibit RNA polymerase II or RNA polymerases II and III. RNA polymerase I mediated synthesis of the rRNA precursor was impaired by treatment of the cells with 50 ng/ml actinomycin D for 1 hour. Cells were fixed and 3D immuno-FISH experiments were performed. Confocal microscopy and image analysis was performed after 3D FISH experiments as follows: series of optical sections through 3D-preserved nuclei were collected using a Leica TCS SP5 confocal system equipped with a Plan Apo 63×/1.4 NA oil immersion objective and a diode laser (excitation wave length 405 nm) for DAPI, an argon laser (488 nm) for FITC and Alexa 488, a DPSS laser (561 nm) for Cy3, a HeNe laser (594 nm) for Texas Red and a HeNe laser (633 nm) for Cy5. For each optical section, signals in different channels were collected sequentially. Stacks of 8-bit gray-scale images were obtained with z-step of 200 nm and pixel sizes 30–100 nm depending on experiment. The axial chromatic shift was corrected and corresponding RGB-stacks, montages and maximum intensity projections were created using published ImageJ plugins [51]. Positions of FISH signals were assessed by visual inspection of RGB stacks using the ImageJ program.

## Supporting Information

Figure S1 Controls of nucleolus purification. Left panel: differential interference contrast (DIC) micrographs show formaldehyde cross-linked HeLa cells and isolated nucleoli. Right panel: UBF and laminA/C immunoblot controls of a nucleolus preparation. Lane 1 shows the input, 2 and 3 the supernatants of the two-step purification [47] and 4 the nucleolar fraction. 0.5% of each fraction was loaded. Quantitative PCR measurement illustrates the enrichment of ribosomal DNA in nucleolus-associated DNA (naDNA) compared to genomic DNA (gDNA). Mean and standard deviation values of two biological replicate experiments are shown.
(0.04 MB PDF)

Figure S2 Nucleolus association maps on all chromosomes detected with 454 sequencing and/or aCGH analysis. 454 and aCGH signals are marked by red and blue bars, 454 and aCGH detected NADs by red and blue rectangles, respectively.
(1.73 MB JPG)

Figure S3 Linear map of NADs and their typical genomic features on the human genome. NADs and their selected, typical sequence features are shown on the map. BAC clones used in 3D FISH experiments are indicated on the top and LADs on the bottom over the Segmental Duplication track. Abbreviations: UR NADs - nucleolus-associated chromosome domains identified in this study, PolI pseudo - pseudogenes of RNA polymerase I transcribed rRNA genes, D4Z4 - D4Z4 major satellite repeats (see Table S4 for further information), OR - olfactory receptor genes, ZNF - zinc finger genes, DEF - defensin genes, 5S and tRNA - 5S rRNA and transfer RNA genes (and pseudogenes) transcribed by RNA polymerase III, NKI LADs - lamin-associated chromosome domains identified by Guelen et al., [21]. Immunoglobulin and T-cell receptor gene clusters are shown according to www.imgt.org [22]. For better view use >400% zoom. Segmental duplications are shown with the colour code identical to the UCSC Genome Browser (http://genome.ucsc.edu/).
(0.92 MB PDF)

Figure S4 Biological processes associated with NAD-located RefSeq genes. Statistical analysis of feature enrichment compared to the genome was performed using the FatiGO strategy [48] included in the Babelomics suite (www.babelomics.org). Enrichment of different features is indicated in red. Statistical values are listed in Table S3. For better view use >300% zoom.
(0.19 MB PDF)

Figure S5 Molecular functions associated with NAD-located RefSeq genes. Statistical analysis of feature enrichment compared to the genome was performed using the FatiGO strategy [48] included in the Babelomics suite (www.babelomics.org). Enrichment of different features is indicated in red. Statistical values are listed in Table S3.
(0.18 MB PDF)

Figure S6 Satellite repeats in NADs and naDNA. The upper panel shows the number of different satellite repeats located in NADs compared to the genomic values. Repeat counts of 454 sequence reads shown in the lower panel reveal other quantitative aspects of different satellite repeat constitution to naDNA. Notably, satellite repeats located on the p-arms of the five acrocentric chromosomes (13, 14, 15, 21, and 22) are not included in the NAD analysis, but they appear in the naDNA analysis. Stars indicate repeats of which substantial amount (30%–50%) is located on chromosome Y and thus missing from female HeLa cells.
(0.13 MB PDF)

Figure S7 2D FISH analysis of BAC clones on human female lymphocyte and HeLa metaphase spreads. Lymphocytes are shown on the left and HeLa on the right panels. DAPI counterstaining is shown in red, BAC hybridization in green. White arrowheads point to BAC signals. Chromosomal localisation was verified by using chromosome paints (not shown). ID codes, chromosomal locations and BACPAC ID numbers of the BACs are indicated. Genomic coordinates of all BACs are shown in Table S7, locations in Figure S3. All BAC clones delivered 2 signals in lymphocytes, but RP11-89H10. However, cross-reaction signals could be filtered since they were significantly less intense than the specific signals. BAC clones delivered 3 signals in HeLa except RP11-89H10, RP11-89O2, RP11-434B14 (2 signals) and RP11-173M10 (4 signals). Again, cross-reaction signals could be filtered in the case of RP11-89H10.
(0.13 MB PDF)

Figure S8 Frequency of nucleolar localisation of NADs and control chromosomal regions detected by 3D FISH in HeLa cervix carcinoma and IMR90 diploid fibroblast cells. Percentage of cells containing at least one nucleolar-localised allele is shown. The results complement the data shown in Figure 4A and summarised in Table S7.
(0.03 MB PDF)

Figure S9 3D immuno-FISH analysis of NADs after inhibition of transcription. HeLa cells were treated with α-amanitin in order to inhibit RNA polymerase II (Pol II) or RNA polymerases II and III (Pol II+III). RNA polymerase I (Pol I) mediated synthesis of the rRNA precursor was impaired by treatment of the cells with actinomycin D. Histograms show the frequency of the nucleolar localisation of three chromosomal regions detected by the indicated BAC clones in 3D FISH experiments. Red, green and blue diamonds indicate target, negative control, and the 5S cluster positive control, respectively (see Table S7 for further BAC details). We used α-amanitin to block transcription by RNA polymerases II and III as described [Huang S, Deerinck TJ, Ellisman MH, Spector DL (1998) The perinucleolar compartment and transcription. J Cell Biol 143: 35–47.; Wang C, Politz JC, Pederson T, Huang S (2003) RNA polymerase III transcripts and the PTB protein are essential for the integrity of the perinucleolar compartment. Mol Biol Cell 14: 2425–2435.], whereas the synthesis of the 47S rRNA precursor was repressed by the addition of actinomycin D as described in the related nucleolar proteome study [19] and in the Materials and Methods. The results show that the specific inhibition of any of the RNA polymerases results in spatial reorganisation of NADs, which indicates that the nucleolus forms a functional unit together with the associated perinucleolar chromatin. Notably, the structure of nucleoli is also partially disrupted after the indicated treatments [38] and thus the interpretation of such analyses is difficult. The results of these experiments are summarised in Table S7.
(0.03 MB PDF)

Figure S10 Quantitative immunofluorescence analysis of selected NAD features. α-H3K27Me3 and α-active Pol II immunostainings of HeLa and IMR90 cells were quantified around nucleoli by using the ImageJ software. After thresholding α-B23/nucleophosmin signals (indicated in blue), mean fluorescence intensity values were measured in the first 250 nm shell (red) and the second 250 nm shell (green) of 12 HeLa cells (22 nucleoli) and 16 IMR90 cells (56 nucleoli). The mean fluorescence intensity values were then divided to estimate enrichment or depletion. At the border of the nucleolus active Pol II and H3K27me3 show a clearly different distribution (p<0.001, Student's t-test). Enrichment and depletion of the two markers in individual shells are significant in all cases (at least at the level p<0.05). Error bars are 95% confidence intervals.
(0.99 MB PDF)

Figure S11 Ribosomal DNA in 454 sequence reads. The assembly of rDNA containing 454 sequence reads is shown in the upper part and the scheme of the rDNA repeat unit below (black arrows indicate the position and direction of individual reads). In total 3,231 rDNA containing DNA fragments were sequenced, of which 2,086 reads were assembled together with the rDNA repeat unit into a single sequence in a MacVector Assembly Project. The results clearly show that different regions were unequally represented in the deep sequencing data, which is probably due to the technical limitations of the method (i.e. emPCR-based amplification of fragments with different GC content is unequal). The negative correlation between the number of sequence reads and GC content can be easily visualized by comparing the assembly result with the GC content plot over the rDNA sequence (the plot was calculated with the EMBOSS Isochore program, http://www.ebi.ac.uk/Tools/emboss). The scheme of the rDNA repeat is shown at the bottom of the figure, 18S, 5.8S, 28S, and IGS mark the coding regions and the intergenic spacer of the human rDNA (GenBank AccNo: U13369), respectively; red and blue lollipops mark the transcriptional start and stop sites, respectively; ticks on the ruler indicate 1 kb distances. We would like to underline here again that the combination of two high-throughput methods, i.e. 454 and aCGH, allows to reduce technical problems, such as the bias in next-generation sequencing [Harismendy O, Ng PC, Strausberg RL, Wang X, Stockwell TB, et al. (2009) Evaluation of next generation sequencing platforms for population targeted sequencing studies. Genome Biol 10: R32.] and the lack of repetitive sequence information in the microarray-based method.
(0.30 MB PDF)

Table S1 List of NAD genomic coordinates (hg18 genome build) and features of their detection. Chromosomal positions and size of NADs is shown in the table. The method of the detection for each 97 NADs is also indicated: 41 NADs were detected with both microarray and high-throughput sequencing, 20 NADs only by using sequencing, and 36 NADs only on microarrays. The number of 454 sequence hits per NAD is shown as well. The 454-based NAD determination was tested in an experimental statistical test comparing the number of reads in each of the detected NADs against the distribution of number of reads in 1,000 randomly selected same-chromosome regions of the same size. The significance is then obtained as the quartile position of the NAD reads number in the random distribution. NADs that were analysed in 3D FISH experiments are highlighted in yellow.
(0.03 MB XLS)

Table S2 List of RefSeq genes located in NADs. Genes within NADs were identified with the UCSC Table Browser (RefSeq Genes Track, hg18 genome build). Note, that almost 30% of the genes are duplicated or even more amplified. Specific enrichment of different gene families in NADs is shown in Figure 3A.
(0.39 MB XLS)

Table S3 Biological processes and molecular functions associated with NAD-located RefSeq genes. Statistical analysis of feature enrichment compared to the genome was performed using the FatiGO strategy [48] included in the Babelomics suite (www.babelomics.org). Results are summarised in Figure S3 and S4 as graphs.
(0.02 MB XLS)

Table S4 List of D4Z4 major satellite containing chromosomal regions of the hg18 genome build. BLAT search was performed using the HUMFSHD sequence (GenBank Accession: D38024) as query. Chromosomal regions with more than 10% (330 bp) homology were indicated on the NAD map (Figure S3).
(0.02 MB XLS)

Table S5 Statistical analysis of sequence features of NADs. Sequence features in NADs, genome and LADs were extracted from the UCSC Table Browser. Fisher's exact test was performed to assess the significance of feature enrichments and the p-values are indicated. One-sided Fisher's exact test was applied to test enrichment of genes of the selected gene families in NADs over the genome values. Two-sided Fisher's exact test was applied to test enrichment/depletion of RNA genes and repeat families in NADs over the genome values. The statistical analysis of the enrichment of satellite repeats and depletion of SINE and in particular MIR repeats in NADs resulted in p = 0, thus they are not listed in the table. Although the differences between the observed NAD and genomic frequencies of other repeat types (LINE, Alu, LTR, DNA; p<<0.001) were also significant, the absolute differences were in these cases smaller than for satellites and MIRs and thus it is less likely that the latter repeats could possess specific nucleolar targeting and/or anchoring potential. The results of gene, RNA gene and repeat content analyses are illustrated as graphs in Figure 3A-3C, respectively. The detailed analysis of satellite repeat classes is shown in Figure S6.
(0.02 MB XLS)

Table S6 Statistical analysis of chromatin features of NADs. Chromatin regulatory features in NADs were extracted from Ensembl Functional Genomics (eFG) database using Ensembl Perl API (Ensembl 50). These data were obtained by ChIP-seq analysis of lymphocytes [34]. The numbers indicate sequence reads per Mb. Additionally, gene expression and H3K27Me3 occupancy data for Hela cells were obtained from the Gene Expression Omnibus Database (GSM323148, GSM323149, GSM325898; [35]). The numbers indicate here sequence length occupied by the H3K27Me3 histone mark per Mb and mean values of gene expression in arbitrary units. Enrichment of features was tested by comparing the distribution of feature counts in NADs against the genome mean value using a t-test statistics and adjusting p-values for multiple testing. Importantly, the analysis of HeLa H3K27Me3 and gene expression data reinforces the results obtained from lymphocytes. Genomic and NAD values of functionally characterised, significantly enriched or depleted chromatin marks are shown in Figure 3D.
(0.05 MB XLS)

Table S7 Summary of 3D FISH experiments. BAC locations, allele and cell counts, furthermore nucleolus association frequencies in HeLa and IMR90 cells are shown. The results of transcription inhibition experiments are summarised in the lower part of the table and illustrated in Figure S9.
(0.02 MB XLS)
