# Supplementary figures and images for: Initial Genomics of the Human Nucleolus
Source: PLoS Genet. 2010 Mar 26;6(3):e1000889. doi: 10.1371/journal.pgen.1000889 (PMC2845662; doi:10.1371/journal.pgen.1000889)

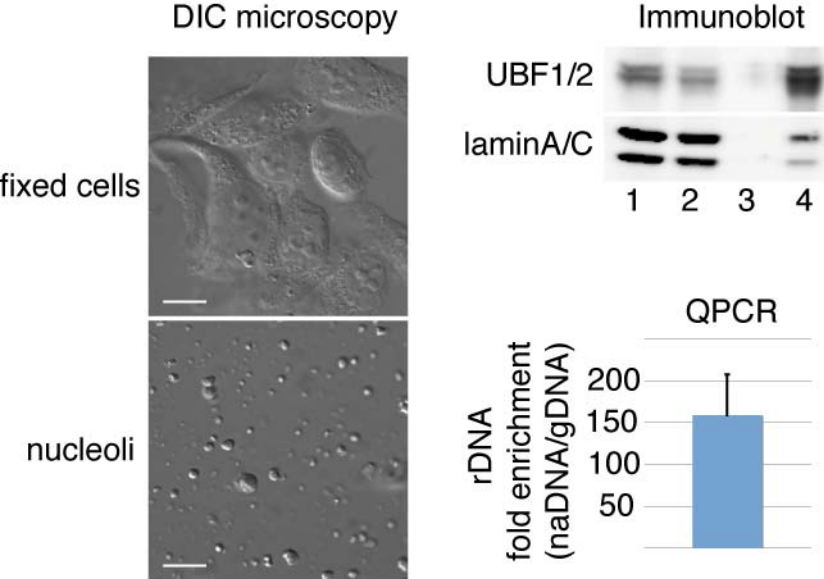

**Fig. S1.**

Supplement: Figure S1 — Controls of nucleolus purification. Left panel: differential interference contrast (DIC) micrographs show formaldehyde cross-linked HeLa cells and isolated nucleoli. Right panel: UBF and laminA/C immunoblot controls of a nucleolus preparation. Lane 1 shows the input, 2 and 3 the supernatants of the two-step purification [47] and 4 the nucleolar fraction. 0.5% of each fraction was loaded. Quantitative PCR measurement illustrates the enrichment of ribosomal DNA in nucleolus-associated DNA (naDNA) compared to genomic DNA (gDNA). Mean and standard deviation values of two biological replicate experiments are shown. (0.04 MB PDF) [file pgen.1000889.s001.pdf]

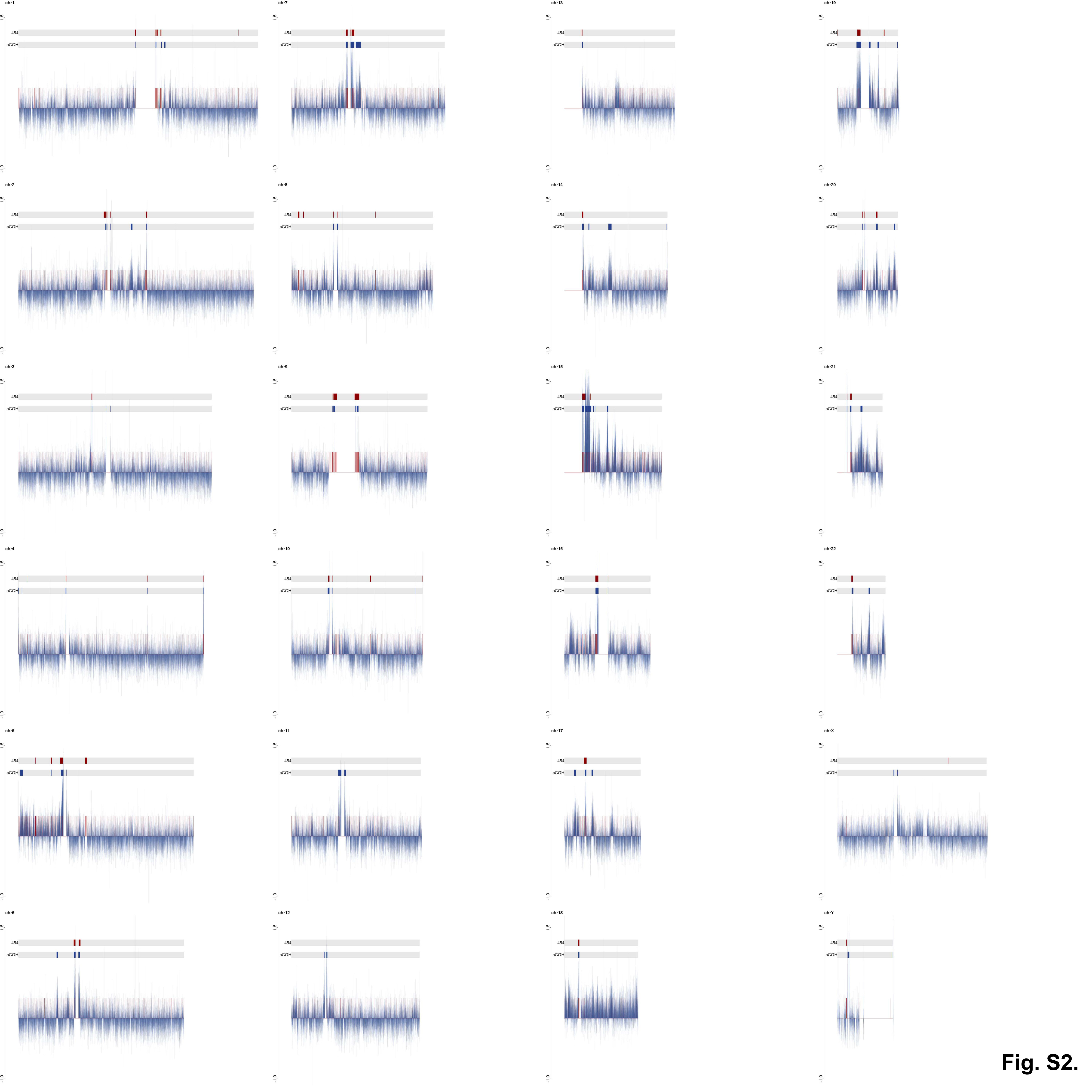

Supplement: Figure S2 — Nucleolus association maps on all chromosomes detected with 454 sequencing and/or aCGH analysis. 454 and aCGH signals are marked by red and blue bars, 454 and aCGH detected NADs by red and blue rectangles, respectively. (1.73 MB JPG) [file pgen.1000889.s002.jpg]

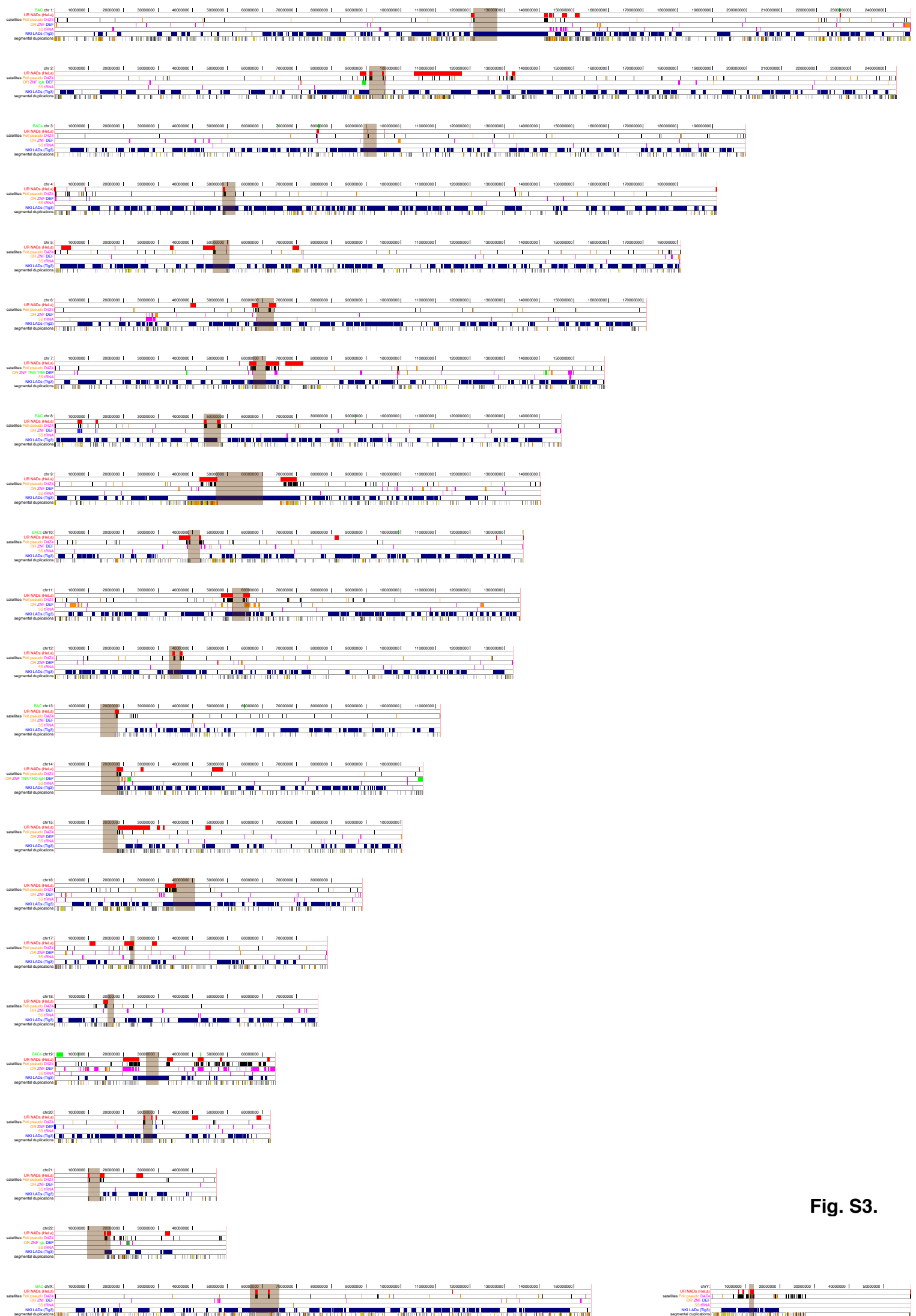

Fig. S3.

Supplement: Figure S3 — Linear map of NADs and their typical genomic features on the human genome. NADs and their selected, typical sequence features are shown on the map. BAC clones used in 3D FISH experiments are indicated on the top and LADs on the bottom over the Segmental Duplication track. Abbreviations: UR NADs - nucleolus-associated chromosome domains identified in this study, PolI pseudo - pseudogenes of RNA polymerase I transcribed rRNA genes, D4Z4 - D4Z4 major satellite repeats (see Table S4 for further information), OR - olfactory receptor genes, ZNF - zinc finger genes, DEF - defensin genes, 5S and tRNA - 5S rRNA and transfer RNA genes (and pseudogenes) transcribed by RNA polymerase III, NKI LADs - lamin-associated chromosome domains identified by Guelen et al., [21]. Immunoglobulin and T-cell receptor gene clusters are shown according to www.imgt.org [22]. For better view use >400% zoom. Segmental duplications are shown with the colour code identical to the UCSC Genome Browser (http://genome.ucsc.edu/). (0.92 MB PDF) [file pgen.1000889.s003.pdf]

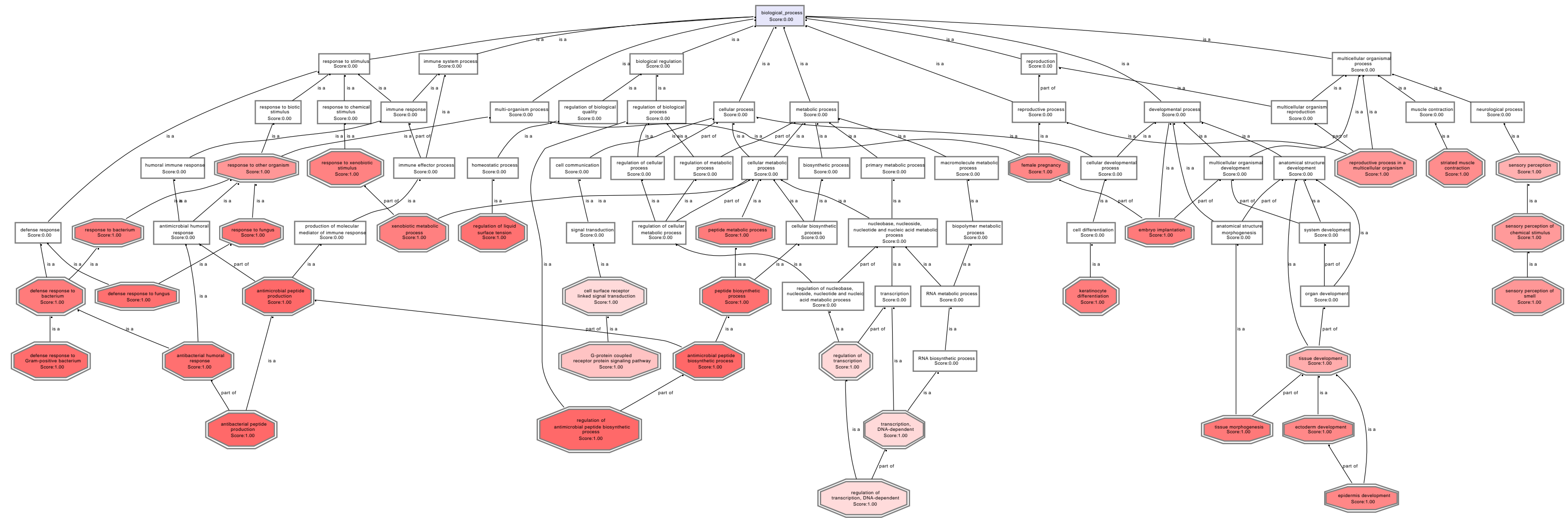

Combined Graph

Fig. S4.

Supplement: Figure S4 — Biological processes associated with NAD-located RefSeq genes. Statistical analysis of feature enrichment compared to the genome was performed using the FatiGO strategy [48] included in the Babelomics suite (www.babelomics.org). Enrichment of different features is indicated in red. Statistical values are listed in Table S3. For better view use >300% zoom. (0.19 MB PDF) [file pgen.1000889.s004.pdf]

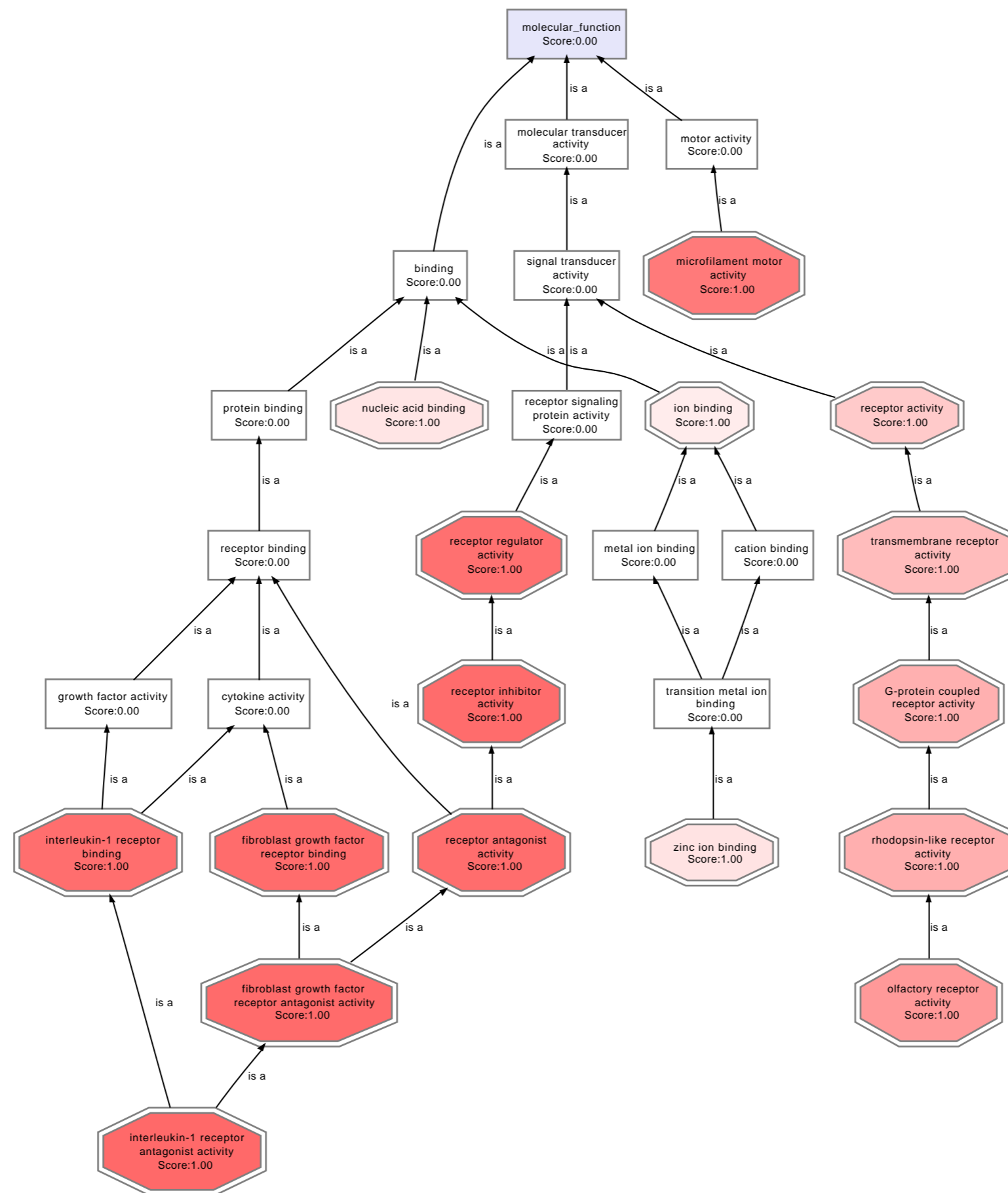

Combined Graph

Fig. S5.

Supplement: Figure S5 — Molecular functions associated with NAD-located RefSeq genes. Statistical analysis of feature enrichment compared to the genome was performed using the FatiGO strategy [48] included in the Babelomics suite (www.babelomics.org). Enrichment of different features is indicated in red. Statistical values are listed in Table S3. (0.18 MB PDF) [file pgen.1000889.s005.pdf]

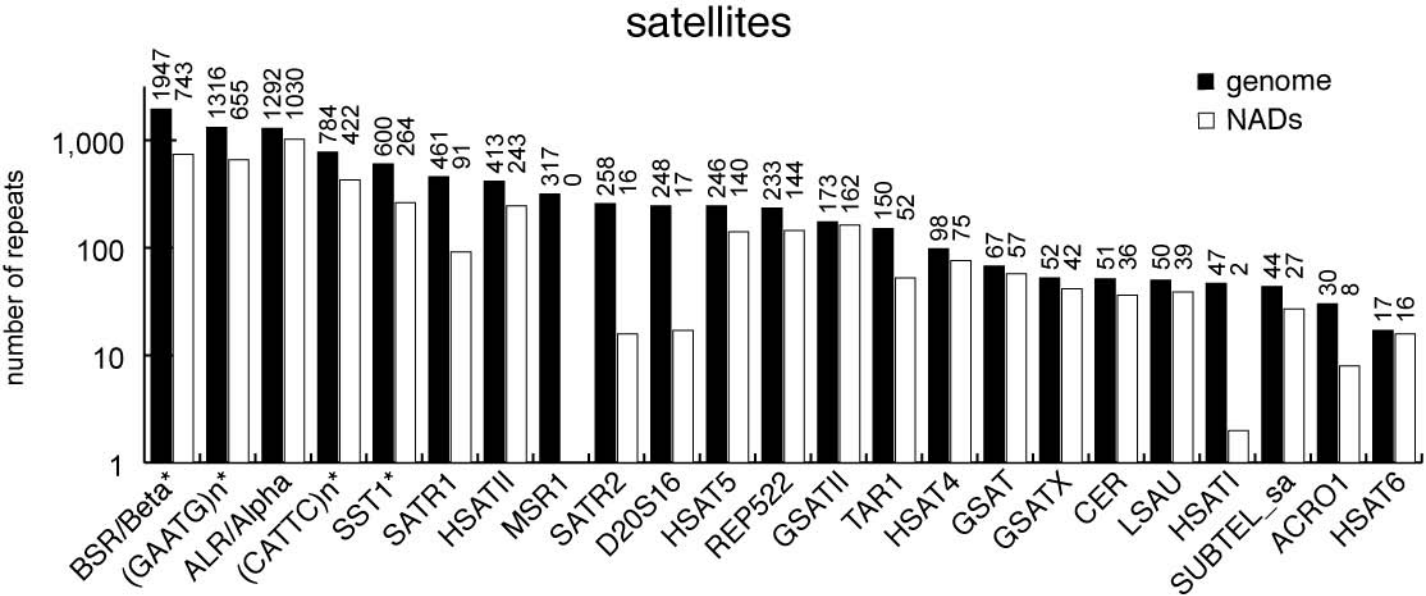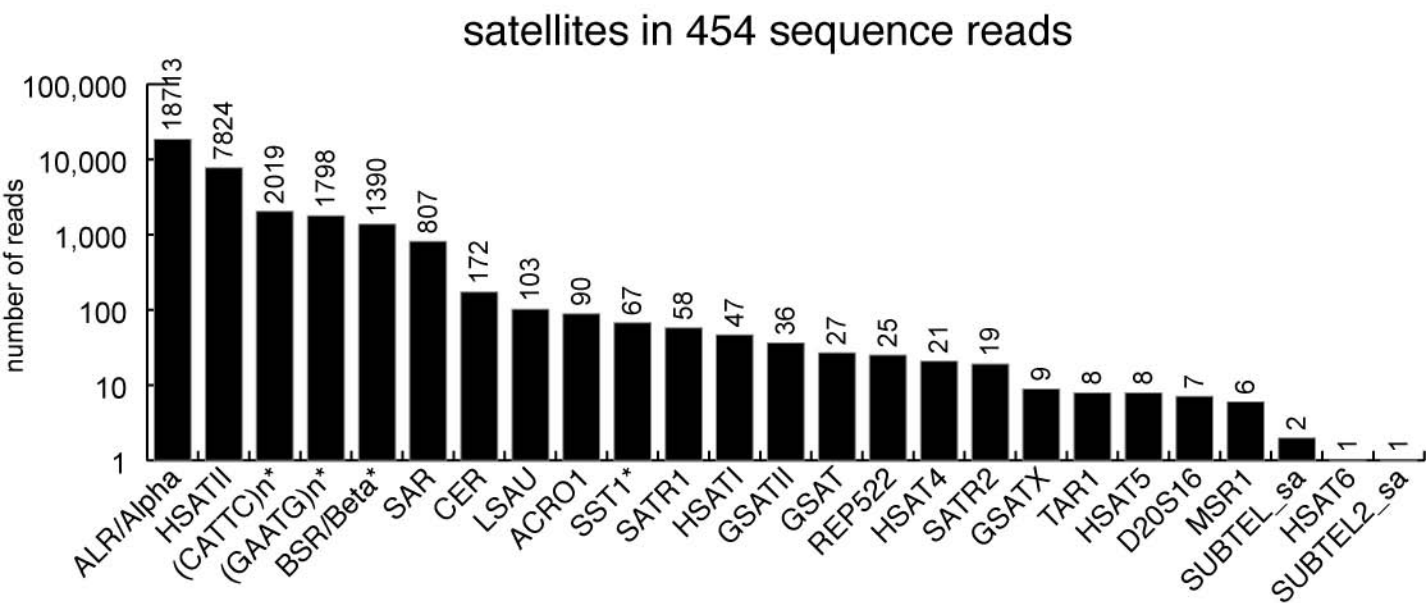

**Fig. S6.**

Supplement: Figure S6 — Satellite repeats in NADs and naDNA. The upper panel shows the number of different satellite repeats located in NADs compared to the genomic values. Repeat counts of 454 sequence reads shown in the lower panel reveal other quantitative aspects of different satellite repeat constitution to naDNA. Notably, satellite repeats located on the p-arms of the five acrocentric chromosomes (13, 14, 15, 21, and 22) are not included in the NAD analysis, but they appear in the naDNA analysis. Stars indicate repeats of which substantial amount (30%–50%) is located on chromosome Y and thus missing from female HeLa cells. (0.13 MB PDF) [file pgen.1000889.s006.pdf]

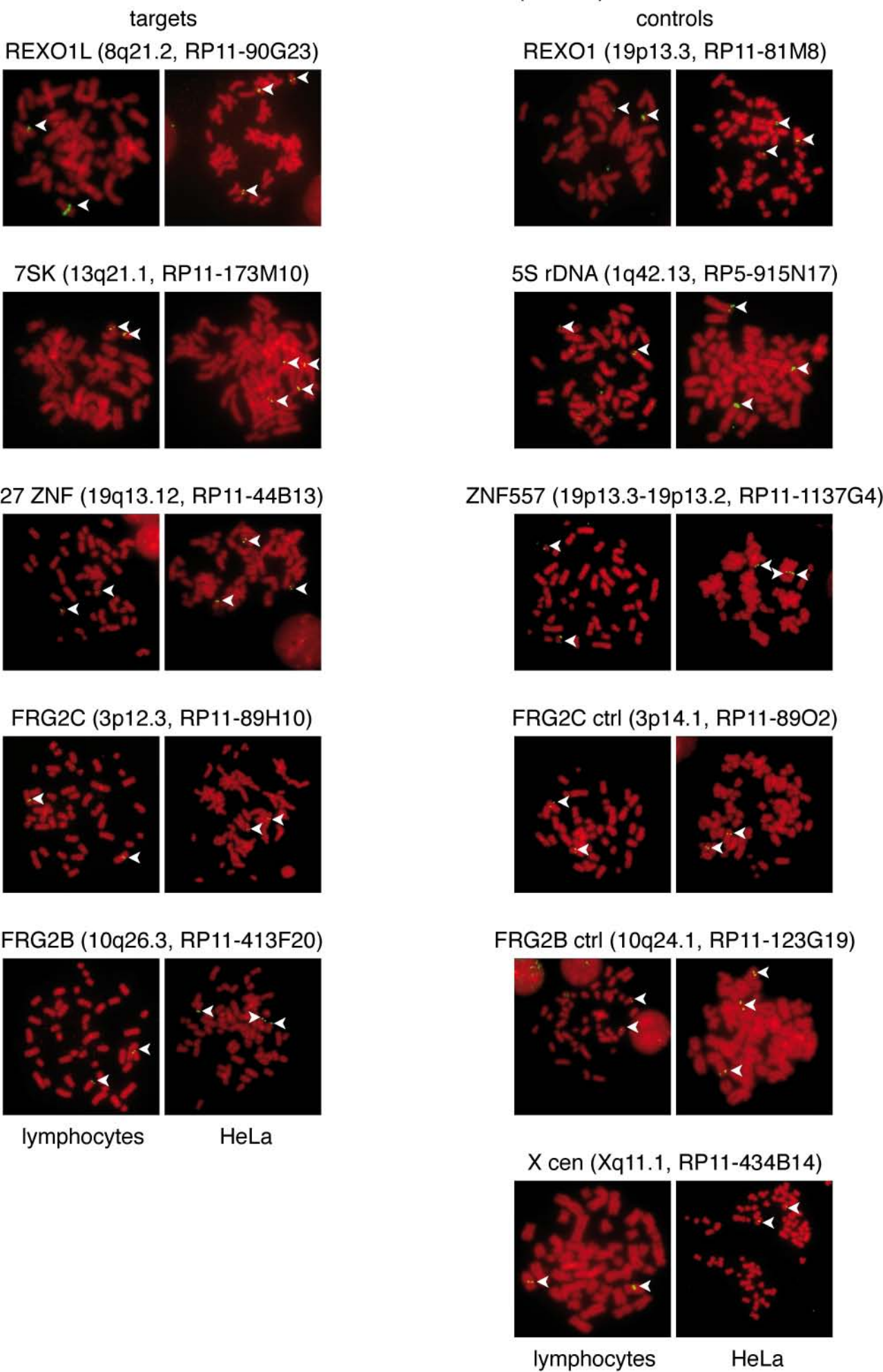

**Fig. S7.**

Supplement: Figure S7 — 2D FISH analysis of BAC clones on human female lymphocyte and HeLa metaphase spreads. Lymphocytes are shown on the left and HeLa on the right panels. DAPI counterstaining is shown in red, BAC hybridization in green. White arrowheads point to BAC signals. Chromosomal localisation was verified by using chromosome paints (not shown). ID codes, chromosomal locations and BACPAC ID numbers of the BACs are indicated. Genomic coordinates of all BACs are shown in Table S7, locations in Figure S3. All BAC clones delivered 2 signals in lymphocytes, but RP11-89H10. However, cross-reaction signals could be filtered since they were significantly less intense than the specific signals. BAC clones delivered 3 signals in HeLa except RP11-89H10, RP11-89O2, RP11-434B14 (2 signals) and RP11-173M10 (4 signals). Again, cross-reaction signals could be filtered in the case of RP11-89H10. (0.13 MB PDF) [file pgen.1000889.s007.pdf]

# 3D-FISH

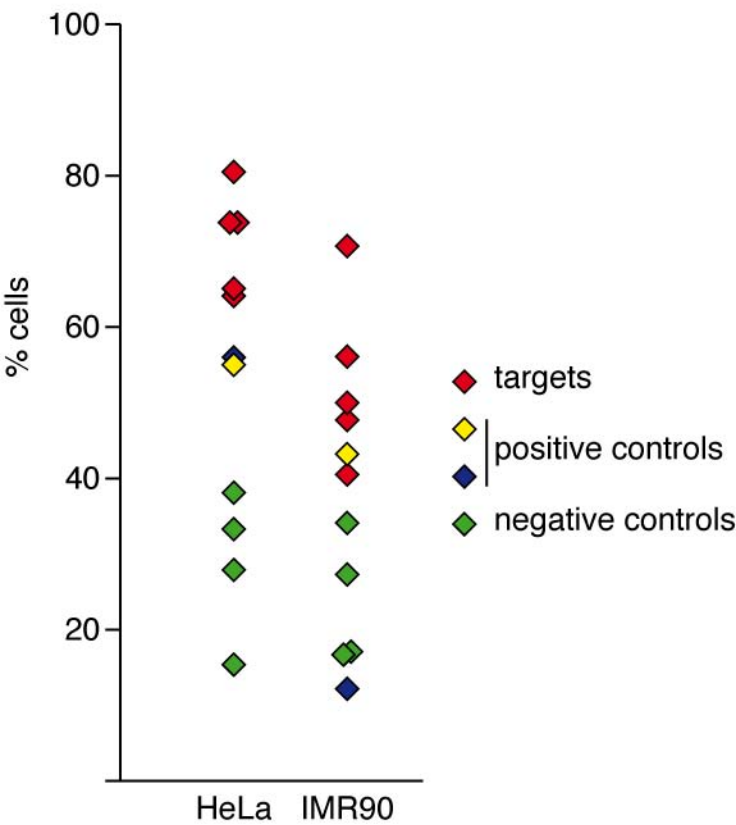

**Fig. S8.**

Supplement: Figure S8 — Frequency of nucleolar localisation of NADs and control chromosomal regions detected by 3D FISH in HeLa cervix carcinoma and IMR90 diploid fibroblast cells. Percentage of cells containing at least one nucleolar-localised allele is shown. The results complement the data shown in Figure 4A and summarised in Table S7. (0.03 MB PDF) [file pgen.1000889.s008.pdf]

### 3D-FISH - drug treatments

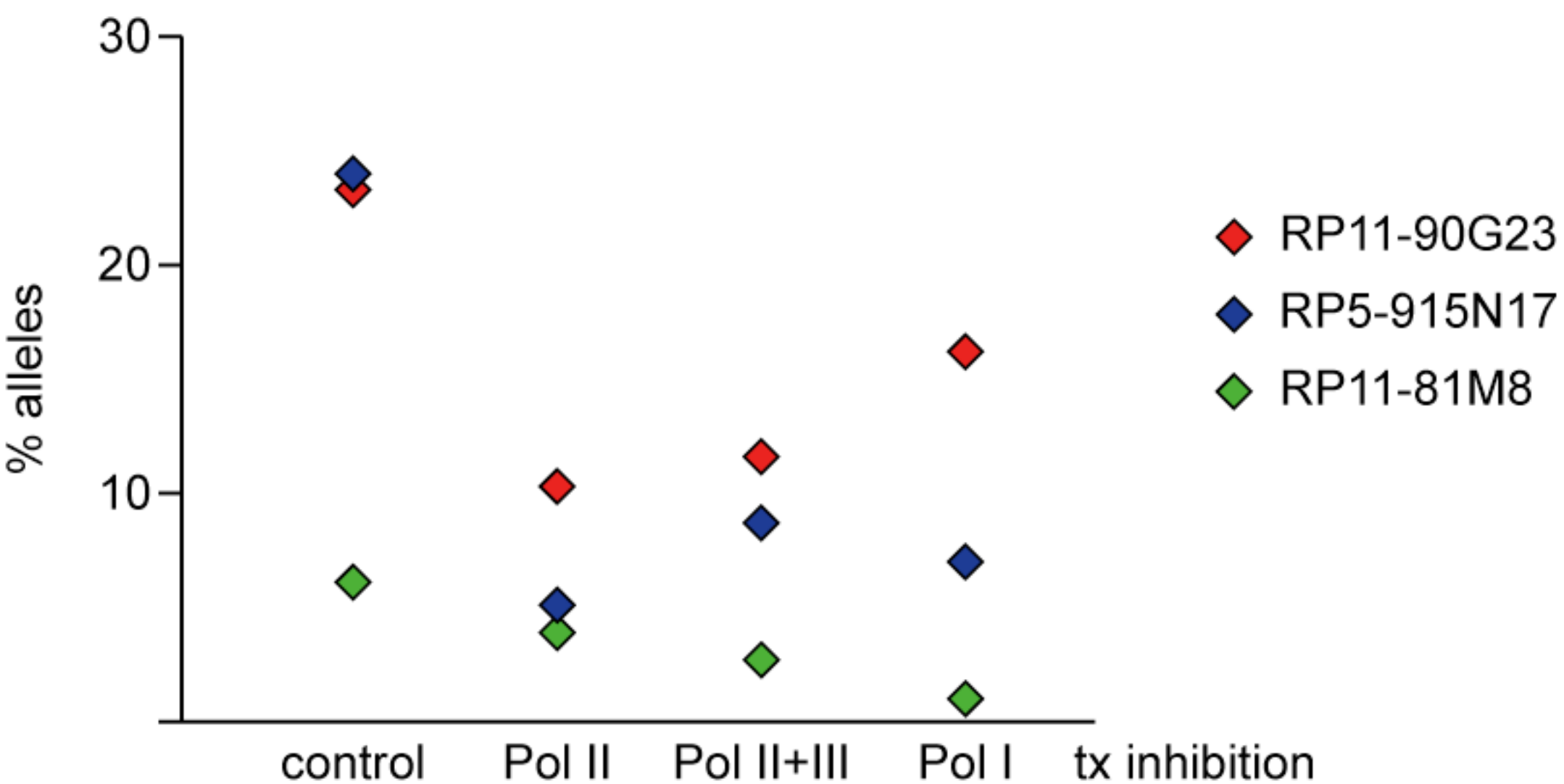

**Fig. S9.**

Supplement: Figure S9 — 3D immuno-FISH analysis of NADs after inhibition of transcription. HeLa cells were treated with α-amanitin in order to inhibit RNA polymerase II (Pol II) or RNA polymerases II and III (Pol II+III). RNA polymerase I (Pol I) mediated synthesis of the rRNA precursor was impaired by treatment of the cells with actinomycin D. Histograms show the frequency of the nucleolar localisation of three chromosomal regions detected by the indicated BAC clones in 3D FISH experiments. Red, green and blue diamonds indicate target, negative control, and the 5S cluster positive control, respectively (see Table S7 for further BAC details). We used α-amanitin to block transcription by RNA polymerases II and III as described [Huang S, Deerinck TJ, Ellisman MH, Spector DL (1998) The perinucleolar compartment and transcription. J Cell Biol 143: 35–47.; Wang C, Politz JC, Pederson T, Huang S (2003) RNA polymerase III transcripts and the PTB protein are essential for the integrity of the perinucleolar compartment. Mol Biol Cell 14: 2425–2435.], whereas the synthesis of the 47S rRNA precursor was repressed by the addition of actinomycin D as described in the related nucleolar proteome study [19] and in the Materials and Methods. The results show that the specific inhibition of any of the RNA polymerases results in spatial reorganisation of NADs, which indicates that the nucleolus forms a functional unit together with the associated perinucleolar chromatin. Notably, the structure of nucleoli is also partially disrupted after the indicated treatments [38] and thus the interpretation of such analyses is difficult. The results of these experiments are summarised in Table S7. (0.03 MB PDF) [file pgen.1000889.s009.pdf]

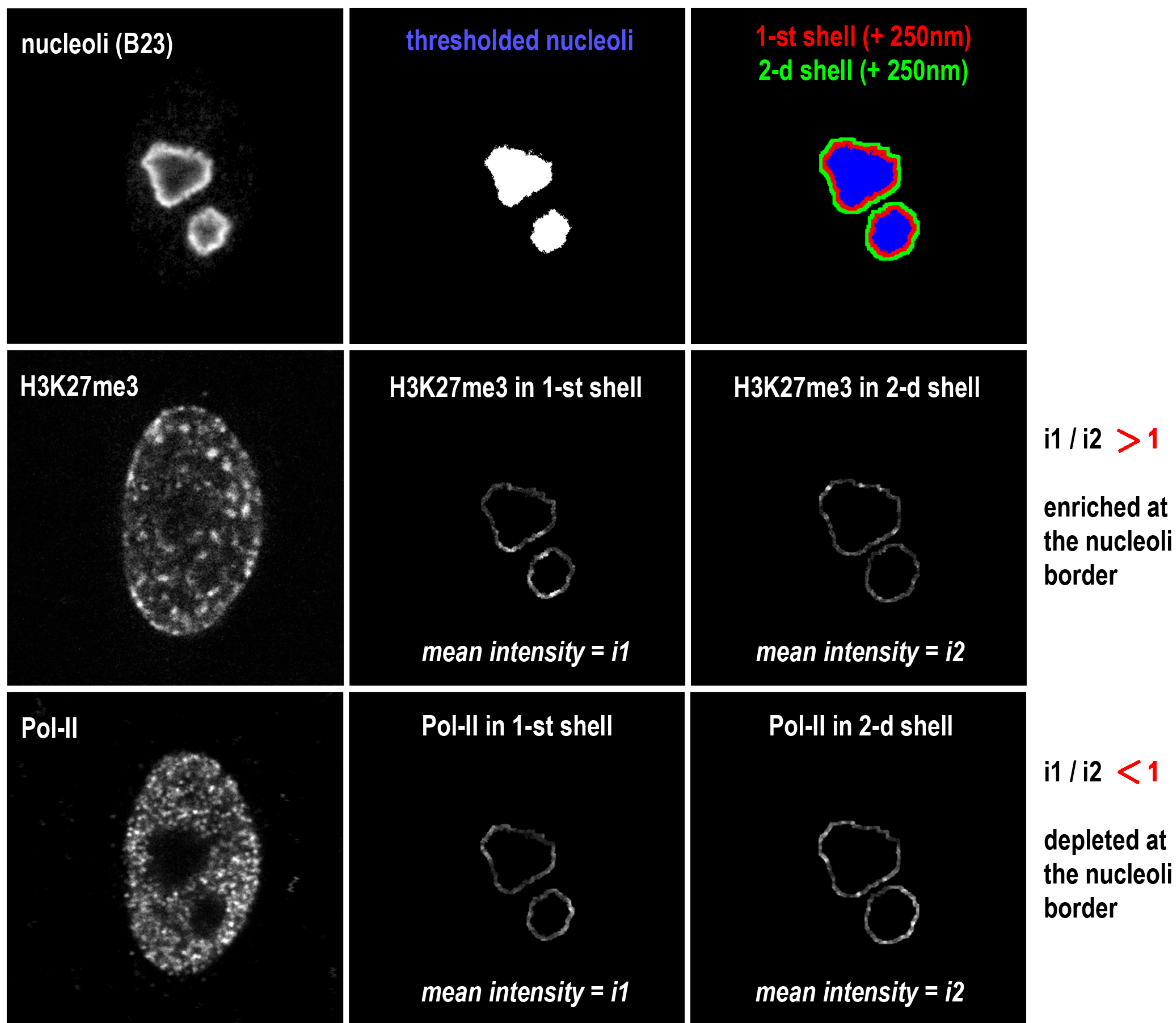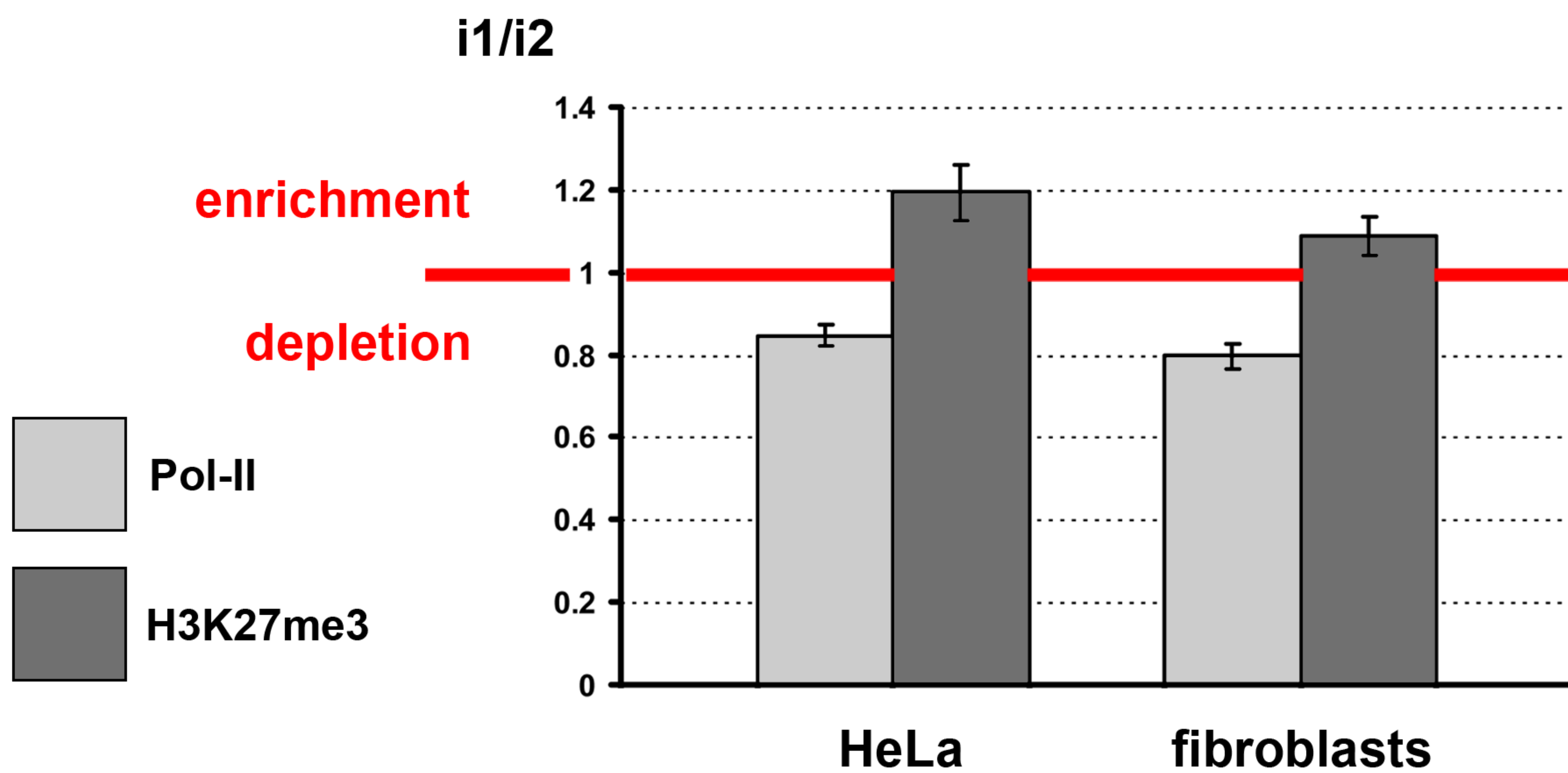

**Fig. S10.**

Supplement: Figure S10 — Quantitative immunofluorescence analysis of selected NAD features. α-H3K27Me3 and α-active Pol II immunostainings of HeLa and IMR90 cells were quantified around nucleoli by using the ImageJ software. After thresholding α-B23/nucleophosmin signals (indicated in blue), mean fluorescence intensity values were measured in the first 250 nm shell (red) and the second 250 nm shell (green) of 12 HeLa cells (22 nucleoli) and 16 IMR90 cells (56 nucleoli). The mean fluorescence intensity values were then divided to estimate enrichment or depletion. At the border of the nucleolus active Pol II and H3K27me3 show a clearly different distribution (p<0.001, Student's t-test). Enrichment and depletion of the two markers in individual shells are significant in all cases (at least at the level p<0.05). Error bars are 95% confidence intervals. (0.99 MB PDF) [file pgen.1000889.s010.pdf]

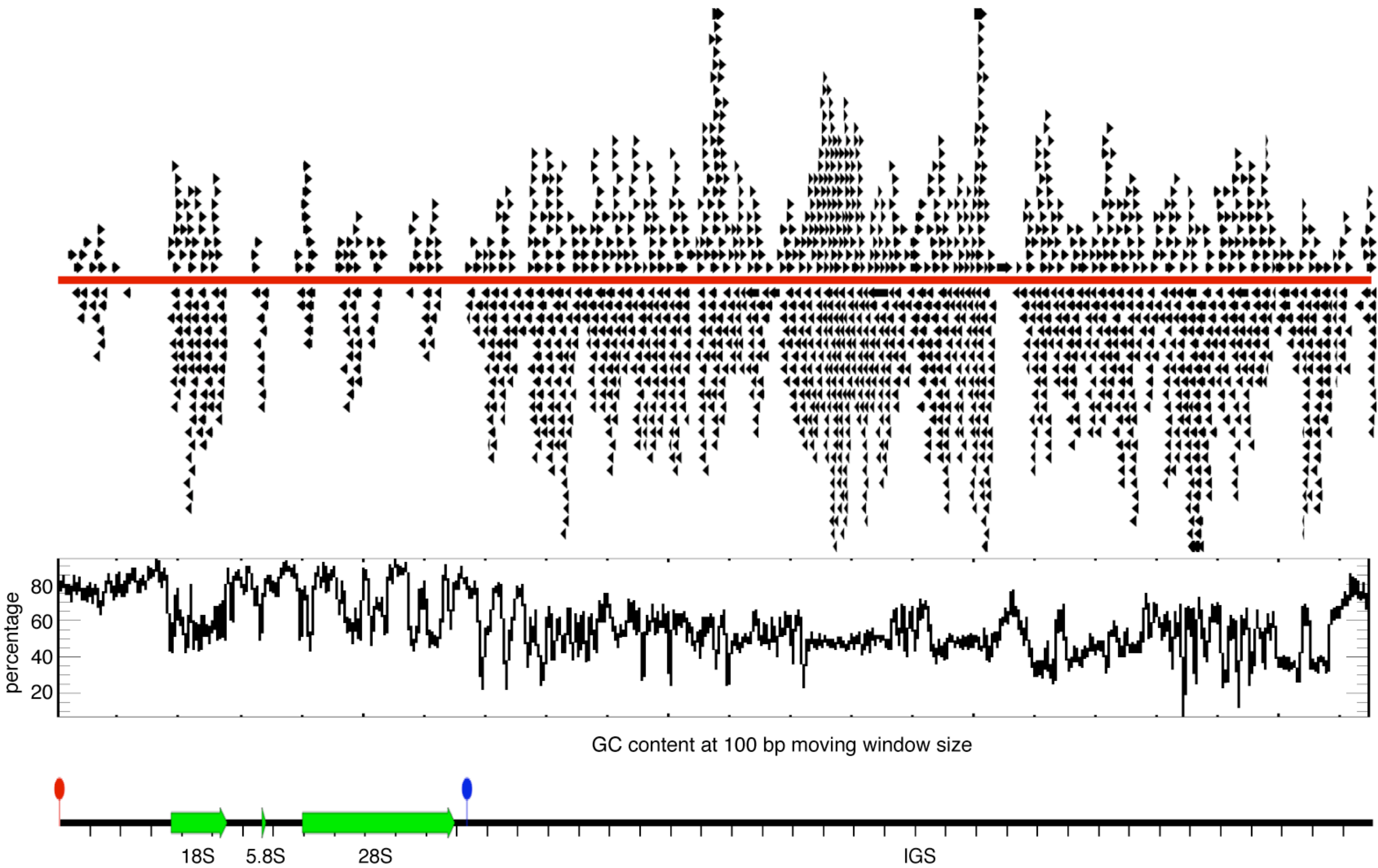

**Fig. S11.**

Supplement: Figure S11 — Ribosomal DNA in 454 sequence reads. The assembly of rDNA containing 454 sequence reads is shown in the upper part and the scheme of the rDNA repeat unit below (black arrows indicate the position and direction of individual reads). In total 3,231 rDNA containing DNA fragments were sequenced, of which 2,086 reads were assembled together with the rDNA repeat unit into a single sequence in a MacVector Assembly Project. The results clearly show that different regions were unequally represented in the deep sequencing data, which is probably due to the technical limitations of the method (i.e. emPCR-based amplification of fragments with different GC content is unequal). The negative correlation between the number of sequence reads and GC content can be easily visualized by comparing the assembly result with the GC content plot over the rDNA sequence (the plot was calculated with the EMBOSS Isochore program, http://www.ebi.ac.uk/Tools/emboss). The scheme of the rDNA repeat is shown at the bottom of the figure, 18S, 5.8S, 28S, and IGS mark the coding regions and the intergenic spacer of the human rDNA (GenBank AccNo: U13369), respectively; red and blue lollipops mark the transcriptional start and stop sites, respectively; ticks on the ruler indicate 1 kb distances. We would like to underline here again that the combination of two high-throughput methods, i.e. 454 and aCGH, allows to reduce technical problems, such as the bias in next-generation sequencing [Harismendy O, Ng PC, Strausberg RL, Wang X, Stockwell TB, et al. (2009) Evaluation of next generation sequencing platforms for population targeted sequencing studies. Genome Biol 10: R32.] and the lack of repetitive sequence information in the microarray-based method. (0.30 MB PDF) [file pgen.1000889.s011.pdf]
